# Postscript

**Published:** 1838-02-28

**Authors:** 


### POSTSCRIPT.

The lecture, delivered by Dr. Gibson, on Saturday, 24th February, he has requested us to report in the present number of the Examiner. As the Doctor intended it as a reply to the clinical remarks of Dr. Harlan, uttered a fortnight previously, and appealed to us to insert it side by side with them, we have given it place, to the exclusion of the foreign and domestic summaries, which were in type. We ask attention to the editorial remarks, which will be found in another column, and venture to express a hope, that we may not have occasion hereafter to exclaim with poor Mercutio, "a plague o' both the houses."

